# Supplementary figures and images for: Endoplasmic reticulum tubules limit the size of misfolded protein condensates
Source: eLife. 2021 Sep 1;10:e71642. doi: 10.7554/eLife.71642 (PMC8486381; doi:10.7554/eLife.71642)

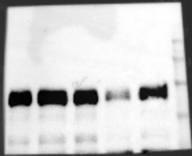

Supplement: Figure 2—source data 1. [file elife-71642-fig2-data1.zip › Figure 2-source data 1-Raw images/SEC24C blot.tif]

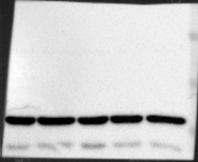

Supplement: Figure 2—source data 1. [file elife-71642-fig2-data1.zip › Figure 2-source data 1-Raw images/GAPDH blot.tif]

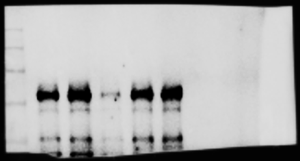

Supplement: Figure 2—figure supplement 1—source data 1. [file elife-71642-fig2-figsupp1-data1.zip › Figure 2-source data 2-Raw images/SEC24B blot.tif]

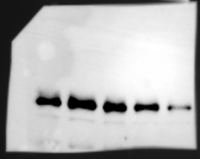

Supplement: Figure 2—figure supplement 1—source data 1. [file elife-71642-fig2-figsupp1-data1.zip › Figure 2-source data 2-Raw images/SEC24D blot.tif]

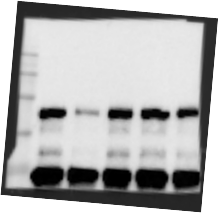

Supplement: Figure 2—figure supplement 1—source data 1. [file elife-71642-fig2-figsupp1-data1.zip › Figure 2-source data 2-Raw images/SEC24A blot.tif]

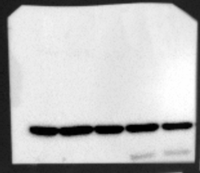

Supplement: Figure 2—figure supplement 1—source data 1. [file elife-71642-fig2-figsupp1-data1.zip › Figure 2-source data 2-Raw images/GAPDH for SEC24D.tif]

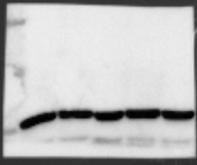

Supplement: Figure 2—figure supplement 1—source data 1. [file elife-71642-fig2-figsupp1-data1.zip › Figure 2-source data 2-Raw images/GAPDH for SEC24A.tif]

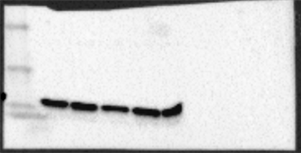

Supplement: Figure 2—figure supplement 1—source data 1. [file elife-71642-fig2-figsupp1-data1.zip › Figure 2-source data 2-Raw images/GAPDH for SEC24B.tif]

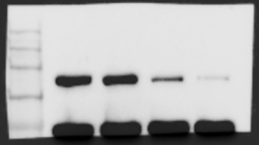

Supplement: Figure 2—figure supplement 1—source data 2. [file elife-71642-fig2-figsupp1-data2.zip › Figure 2-source data 3-Raw images/SEC24C blot.tif]

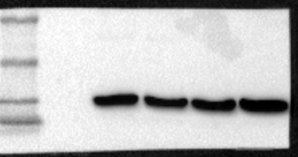

Supplement: Figure 2—figure supplement 1—source data 2. [file elife-71642-fig2-figsupp1-data2.zip › Figure 2-source data 3-Raw images/GAPDH blot.tif]

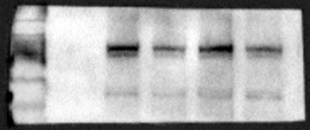

Supplement: Figure 2—figure supplement 1—source data 2. [file elife-71642-fig2-figsupp1-data2.zip › Figure 2-source data 3-Raw images/RTN3 blot.tif]

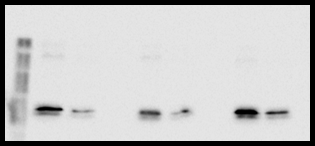

Supplement: Figure 2—figure supplement 1—source data 3. [file elife-71642-fig2-figsupp1-data3.zip › Figure 2-source data 4-Raw images/FAM134B blot.tif]

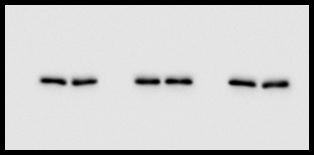

Supplement: Figure 2—figure supplement 1—source data 3. [file elife-71642-fig2-figsupp1-data3.zip › Figure 2-source data 4-Raw images/GAPDH.tif]

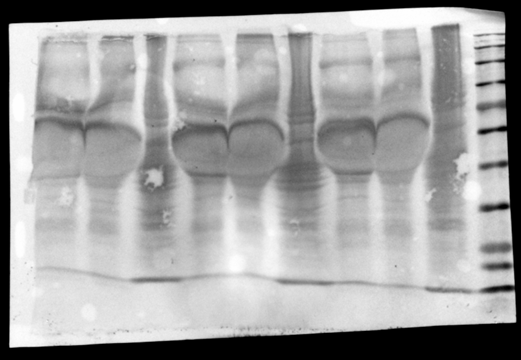

Supplement: Figure 5—figure supplement 5—source data 1. [file elife-71642-fig5-figsupp5-data1.zip › Figure 5-source data 1-Raw images/Ponceau.tif]

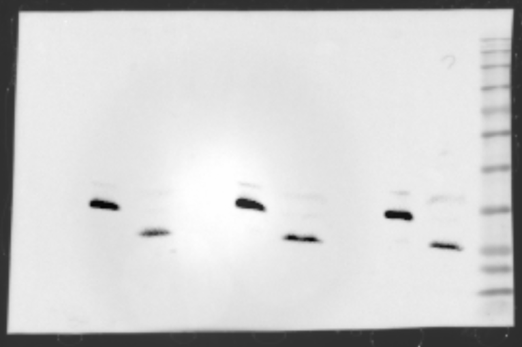

Supplement: Figure 5—figure supplement 5—source data 1. [file elife-71642-fig5-figsupp5-data1.zip › Figure 5-source data 1-Raw images/Cpep GFP blot.tif]

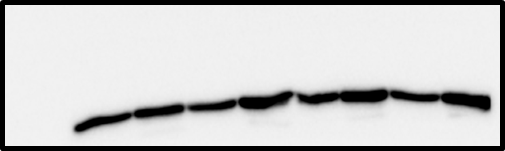

Supplement: Figure 6—source data 1. [file elife-71642-fig6-data1.zip › Figure 6-source data 1-Raw images/GAPDH for RTN3.tif]

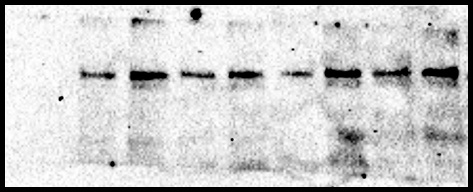

Supplement: Figure 6—source data 1. [file elife-71642-fig6-data1.zip › Figure 6-source data 1-Raw images/RTN3 blot.tif]

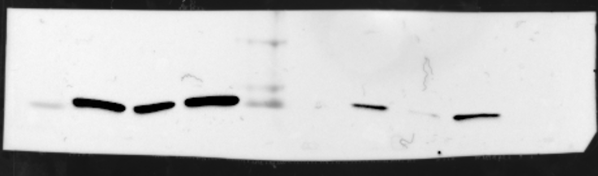

Supplement: Figure 6—source data 1. [file elife-71642-fig6-data1.zip › Figure 6-source data 1-Raw images/GAPDH for LNPK.tif]

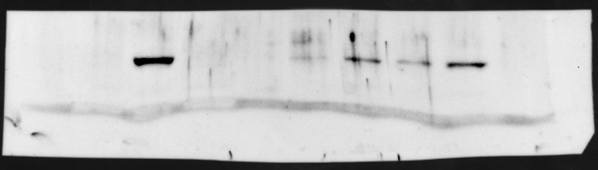

Supplement: Figure 6—source data 1. [file elife-71642-fig6-data1.zip › Figure 6-source data 1-Raw images/LNPK blot.tif]
