# Supplementary material for: Endoplasmic reticulum tubules limit the size of misfolded protein condensates
Source: eLife. 2021 Sep 1;10:e71642. doi: 10.7554/eLife.71642 (PMC8486381; doi:10.7554/eLife.71642)
Supplement: Figure 2—figure supplement 1—source data 1. [file elife-71642-fig2-figsupp1-data1.zip › Figure 2-source data 2.pdf]

A

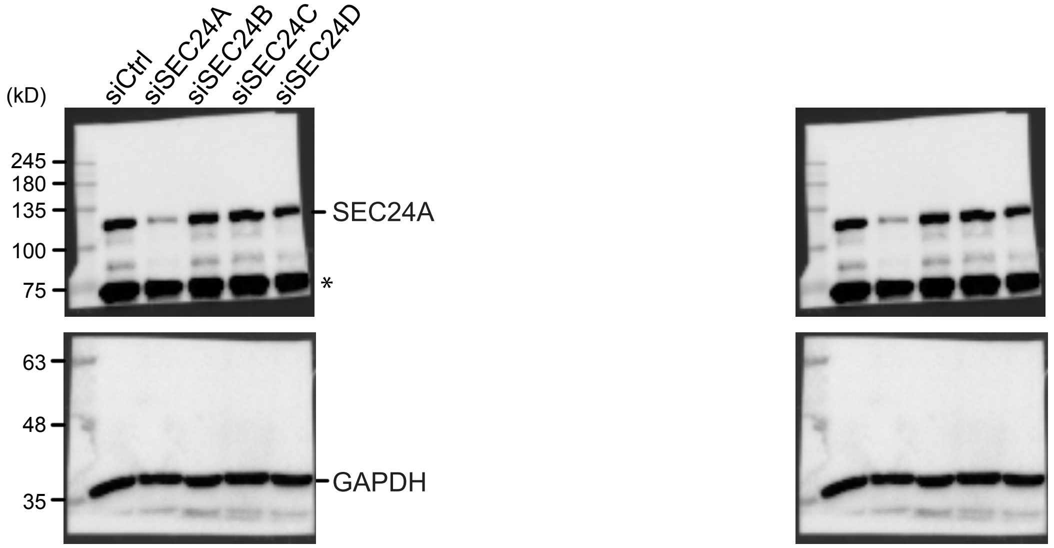

B

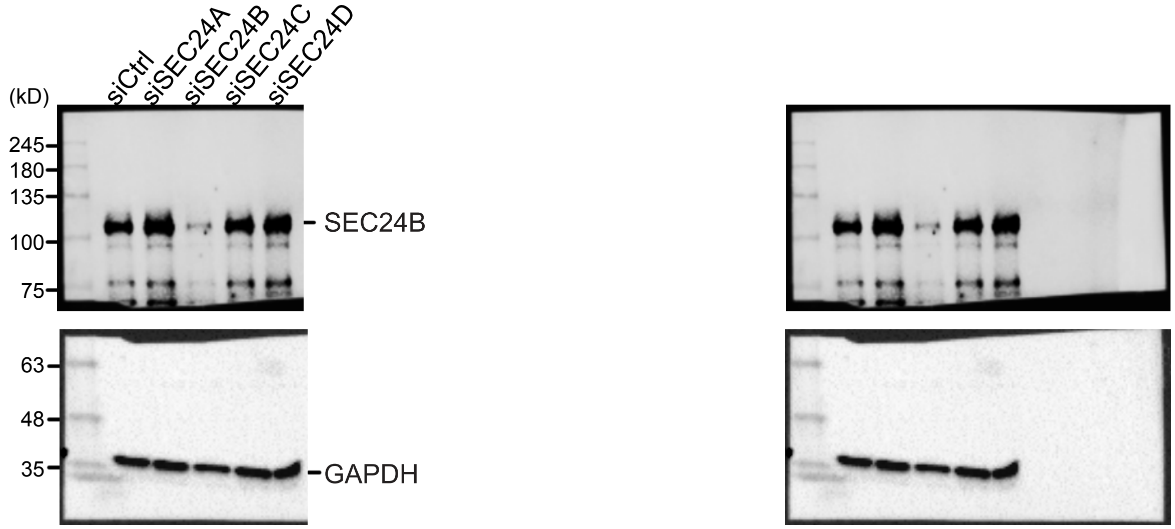

C

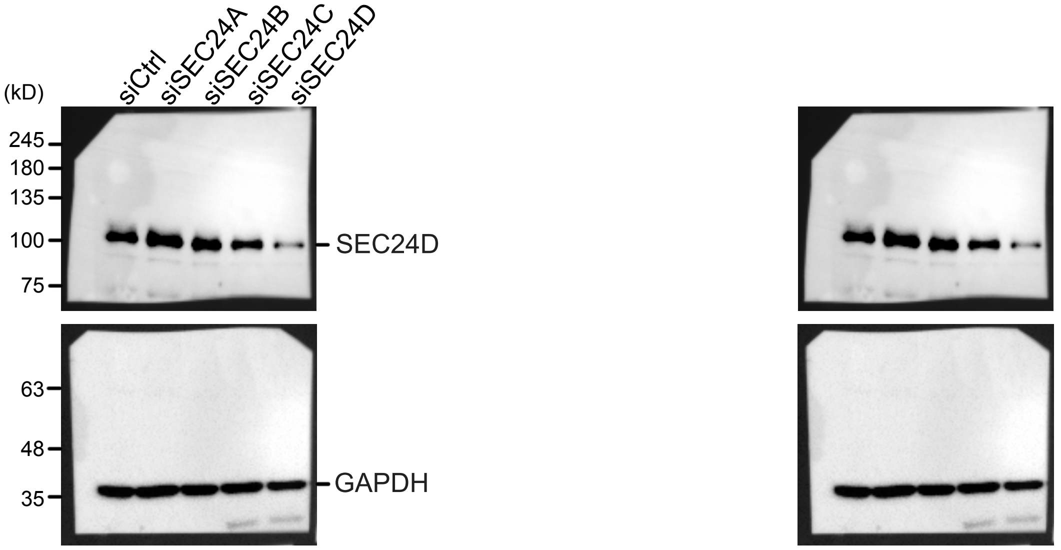

Figure 2-source data 2. Uncropped blots for Figure supplement 1B.
A) Left top, labeled SEC24A blot of uncropped raw blot on the right. Left bottom, labeled GAPDH blot of uncropped raw blot on the right. Asterisk marks non-specific cross reacting band. B) Left top, labeled SEC24B blot of uncropped raw blot on the right. Left bottom, labeled GAPDH blot of uncropped raw blot on the right. C) Left top, labeled SEC24D blot of uncropped raw blot on the right. Left bottom, labeled GAPDH blot of uncropped raw blot on the right.
