# Supplementary material for: Endoplasmic reticulum tubules limit the size of misfolded protein condensates
Source: eLife. 2021 Sep 1;10:e71642. doi: 10.7554/eLife.71642 (PMC8486381; doi:10.7554/eLife.71642)
Supplement: Figure 2—figure supplement 1—source data 2. [file elife-71642-fig2-figsupp1-data2.zip › Figure 2-source data 3.pdf]

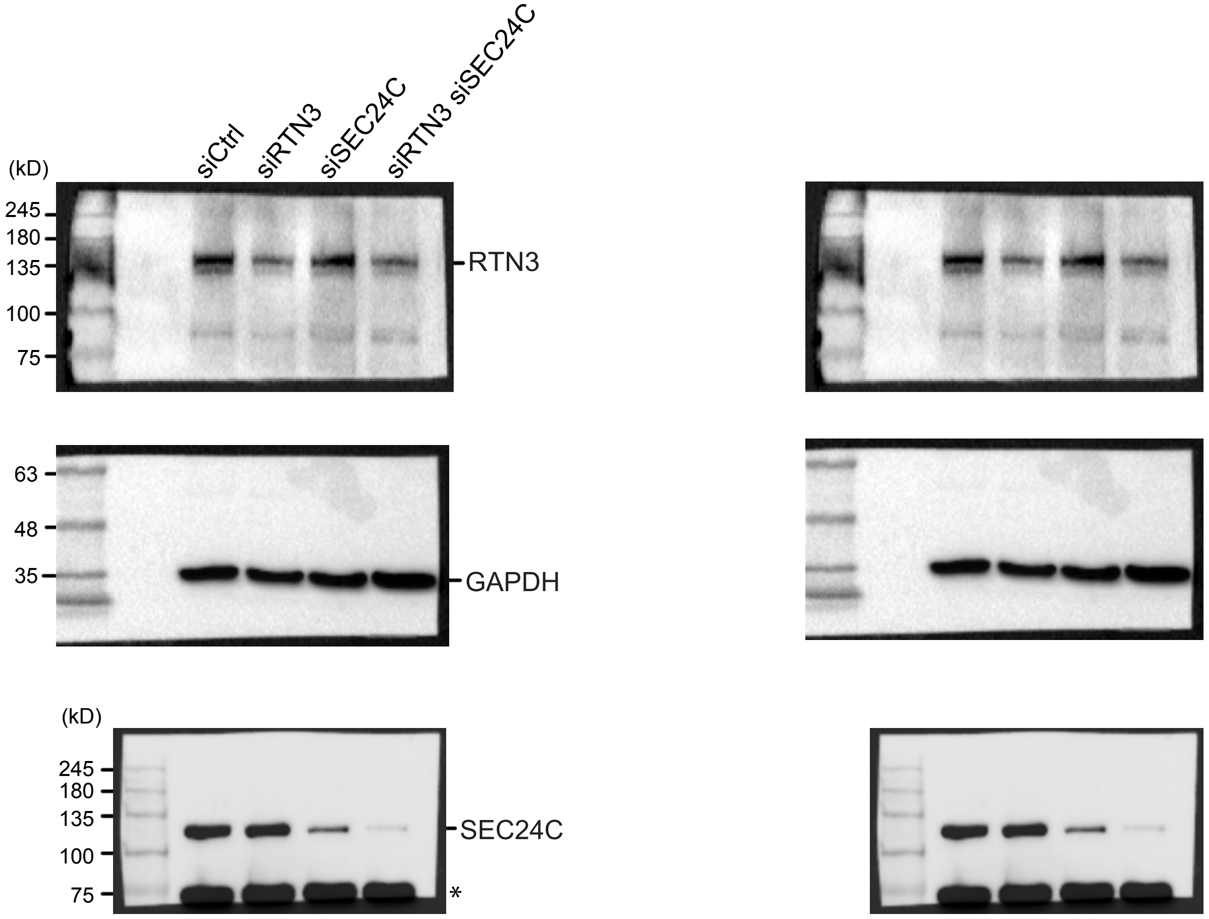

Figure 2-source data 3. Uncropped blots for Figure supplement 1D.
Left top, labeled RTN3 blot of uncropped raw blot on the right. Left middle, labeled GAPDH blot of uncropped raw blot on the right. Left bottom, labeled SEC24C blot of uncropped raw blot on the right. Asterisk marks non-specific cross-reacting band.
