# Supplementary material for: Endoplasmic reticulum tubules limit the size of misfolded protein condensates
Source: eLife. 2021 Sep 1;10:e71642. doi: 10.7554/eLife.71642 (PMC8486381; doi:10.7554/eLife.71642)
Supplement: Figure 2—figure supplement 1—source data 3. [file elife-71642-fig2-figsupp1-data3.zip › Figure 2-source data 4.pdf]

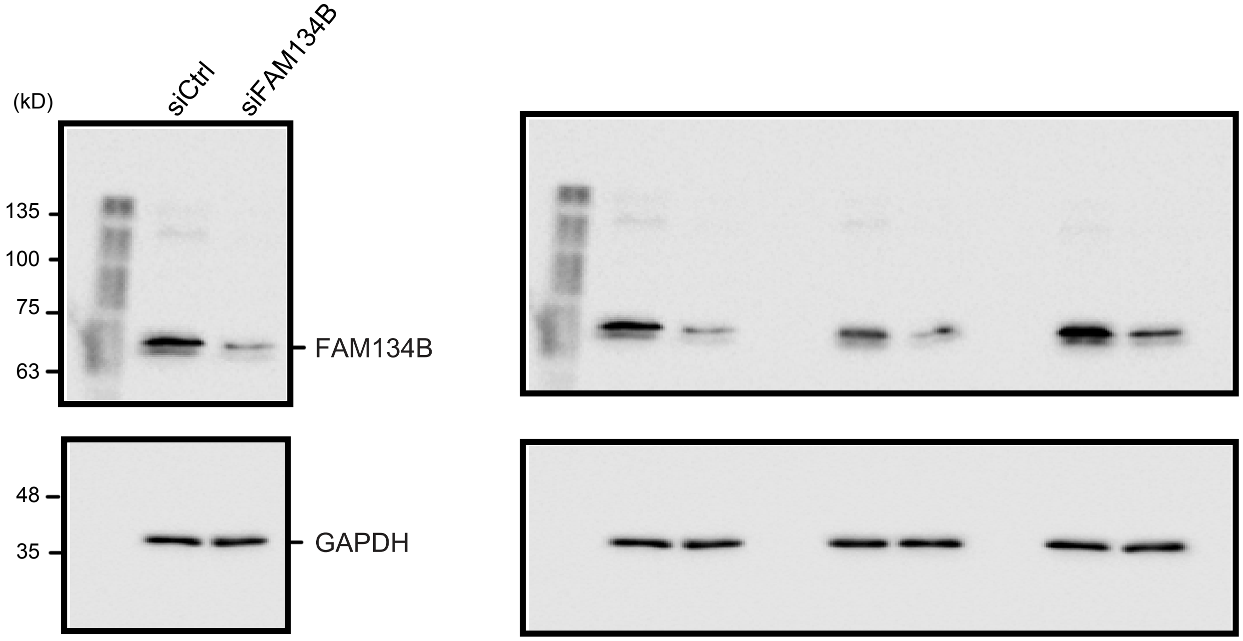


**Figure 2-source data 4. Uncropped blots for Figure supplement 1F.**
Left top, labeled FAM134B blot of uncropped raw blot on the right. Left bottom, labeled GAPDH blot of uncropped raw blot on the right.
