# Supplementary material for: Endoplasmic reticulum tubules limit the size of misfolded protein condensates
Source: eLife. 2021 Sep 1;10:e71642. doi: 10.7554/eLife.71642 (PMC8486381; doi:10.7554/eLife.71642)
Supplement: Figure 5—figure supplement 5—source data 1. [file elife-71642-fig5-figsupp5-data1.zip › Figure 5-source data 1.pdf]

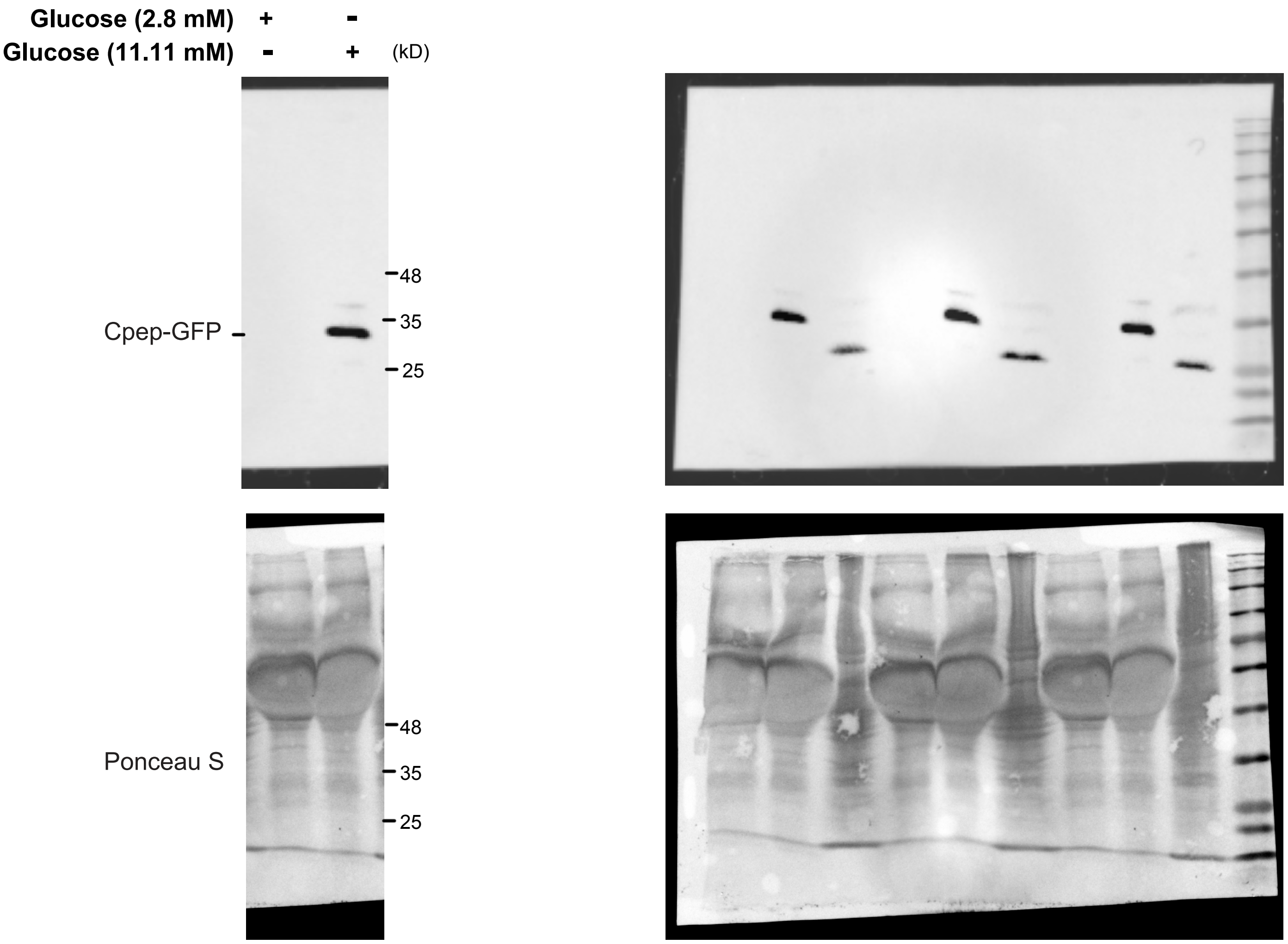

Figure 5-source data 1. Uncropped blots for Figure supplement 5D.
Left top, labeled Cpep-GFP blot of uncropped raw blot on the right. Left bottom, labeled Ponceau S-stained blot of uncropped raw blot on the right.
