# Supplementary material for: Endoplasmic reticulum tubules limit the size of misfolded protein condensates
Source: eLife. 2021 Sep 1;10:e71642. doi: 10.7554/eLife.71642 (PMC8486381; doi:10.7554/eLife.71642)
Supplement: Figure 6—source data 1. [file elife-71642-fig6-data1.zip › Figure 6-source data 1.pdf]

A

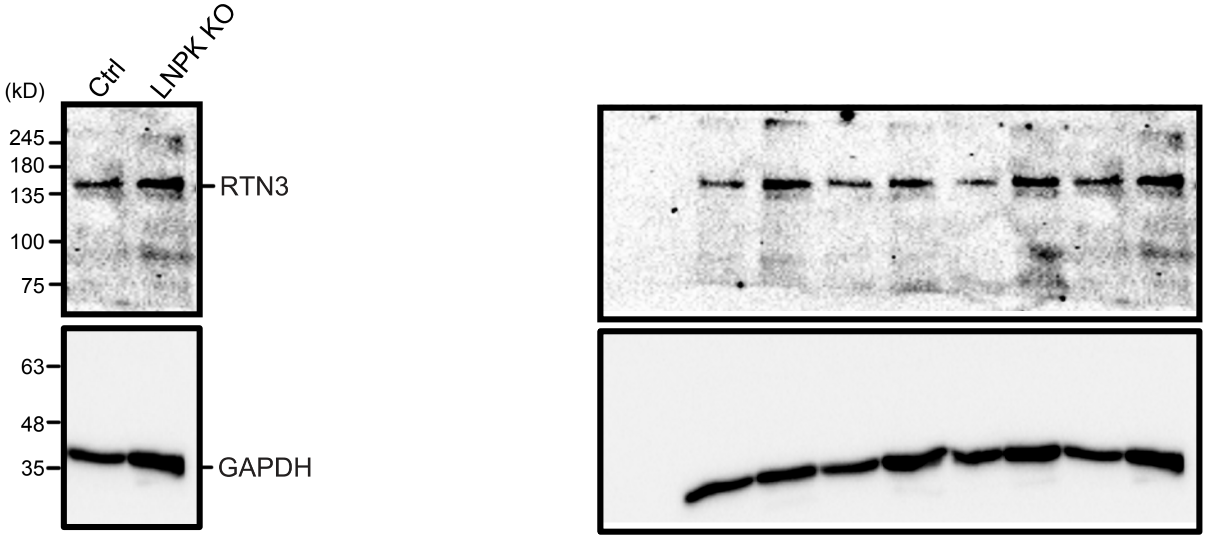

B

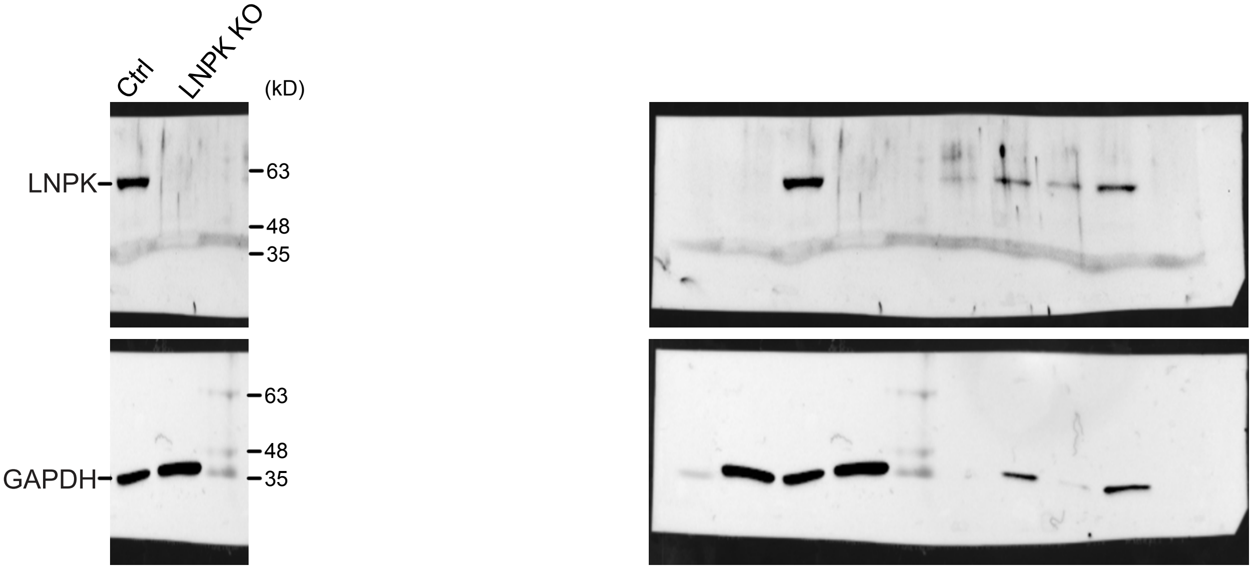

Figure 6-source data 1. Uncropped blots for Figure 6A.

A) Left top, labeled RTN3 blot of uncropped raw blot on the right. Left bottom, labeled GAPDH blot of uncropped raw blot on the right. B) Left top, labeled LNPK blot of uncropped raw blot on the right. Left bottom, labeled GAPDH blot of uncropped raw blot on the right.
